# Global Gene Expression Profiling in PPAR-**γ** Agonist-Treated Kidneys in an Orthologous Rat Model of Human Autosomal Recessive Polycystic Kidney Disease

**DOI:** 10.1155/2012/695898

**Published:** 2012-05-13

**Authors:** Daisuke Yoshihara, Masanori Kugita, Tamio Yamaguchi, Harold M. Aukema, Hiroki Kurahashi, Miwa Morita, Yoshiyuki Hiki, James P. Calvet, Darren P. Wallace, Takafumi Toyohara, Takaaki Abe, Shizuko Nagao

**Affiliations:** ^1^Education and Research Center of Animal Models for Human Diseases, Fujita Health University, Toyoake, Aichi 4701192, Japan; ^2^Division of Molecular Genetics, Institute for Comprehensive Medical Science, Fujita Health University, Toyoake, Aichi 4701192, Japan; ^3^Department of Human Nutritional Sciences, University of Manitoba, Winnipeg, MB, Canada R3T 2N2; ^4^School of Health Sciences, Fujita Health University, Toyoake, Aichi 4701192, Japan; ^5^The Kidney Institute, University of Kansas Medical Center, Kansas City, KS 66160, USA; ^6^Department of Clinical Biology and Hormonal Regulation, Tohoku University Graduate School of Biomedical Engineering, Sendai, Miyagi 9808574, Japan

## Abstract

Kidneys are enlarged by aberrant proliferation of tubule epithelial cells leading to the formation of numerous cysts, nephron loss, and interstitial fibrosis in polycystic kidney disease (PKD). Pioglitazone (PIO), a PPAR-**γ** agonist, decreased cell proliferation, interstitial fibrosis, and inflammation, and ameliorated PKD progression in PCK rats (*Am. J. Physiol*.-*Renal*, 2011). To explore genetic mechanisms involved, changes in global gene expression were analyzed. By Gene Set Enrichment Analysis of 30655 genes, 13 of the top 20 downregulated gene ontology biological process gene sets and six of the top 20 curated gene set canonical pathways identified to be downregulated by PIOtreatment were related to cell cycle and proliferation, including EGF, PDGF and JNK pathways. Their relevant pathways were identified using the Kyoto Encyclopedia of Gene and Genomes database. Stearoyl-coenzyme A desaturase 1 is a key enzyme in fatty acid metabolism found in the top 5 genes downregulated by PIO treatment. Immunohistochemical analysis revealed that the gene product of this enzyme was highly expressed in PCK kidneys and decreased by PIO. These data show that PIO alters the expression of genes involved in cell cycle progression, cell proliferation, and fatty acid metabolism.

## 1. Introduction

Polycystic kidney diseases (PKD) are characterized by progressive enlargement of numerous fluid-filled cysts in both kidneys, often leading to chronic kidney disease (CKD). Autosomal dominant PKD (ADPKD) is one of the most common hereditary disorders in humans with an incidence of 1 : 500–1,000, caused by mutations in the *PKD1* or *PKD2* gene. Progressive kidney enlargement is due to aberrant proliferation of the cystic epithelia, together with an accumulation of fluid into the cyst cavities due to transepithelial chloride (Cl^−^) and fluid secretion [1–3]. Autosomal recessive PKD (ARPKD) is known as a juvenile-type cystic disease with an incidence of 1 : 20,000 [3]. Kidneys in ARPKD patients are characterized by cystic fusiform dilations of the collecting ducts accompanied by increased cell proliferation and fluid secretion, leading to massive kidney enlargement and renal failure occurring in the first few years after birth [4]. Increased cell proliferation, stimulated fluid secretion, and interstitial fibrosis are often observed in cystic liver disease in ARPKD as well [5]. 

Peroxisome proliferator-activated receptors (PPARs) belong to a nuclear receptor superfamily of ligand-activated transcription factors with subtypes *α*, *β*/*δ*, and *γ*. PPAR-*γ* is widely expressed in several organs including kidneys and known to be activated by fatty acids [6, 7]. Antidiabetic agents, pioglitazone (PIO), troglitazone, ciglitazone, and rosiglitazone, are used to control blood sugar levels in patients with diabetes mellitus. These PPAR-*γ* agonists also have important roles in regulation of cell cycle, inhibition of fibrosis, infiltration and metastasis of cancer cells, and modulation of inflammatory cytokines.

Treatment with PIO improved survival and ameliorated cardiac defects and the degree of renal cystogenesis in embryos of *Pkd1^−/−^* mice in a previous study [8]. In addition, long-term treatment of this agonist improved endothelial function by increasing production of nitric oxide in adult heterozygous *Pkd1^+/−^* mice [8]. Another PPAR-*γ* agonist, rosiglitazone attenuated PKD progression and prolonged survival of Han: SPRD Cy rats [9]. In our recent study, daily treatment of PIO ameliorated polycystic kidney disease through inhibiting Raf/MEK/ERK and AKT/mTOR/S6 signaling cascades in the PCK rat, an orthologous model of human ARPKD [10]. These findings suggest that PPAR-*γ* agonists may have therapeutic value in ARPKD via altering several cellular signaling pathways. In the current study, we applied global gene expression profiling to explore novel cellular signaling pathways potentially related to the ameliorating effects of PIO in PCK rat kidneys.

## 2. Methods

### 2.1. PCK Rat and Study Design

PCK rats were originally derived from a strain of Sprague-Dawley rats in Japan and descendants of this colony have been maintained at the Education and Research Center of Animal Models for Human Diseases, Fujita Health University. PCK rats and normal Sprague Dawley rats (+/+; Charles River Japan Inc., Kanagawa, Japan) were allowed free access to water and food throughout the study. Female PCK and +/+ rats, aged 4–20 weeks (*n* = 10 per gender) were randomly assigned to one of two groups: treatment with 10 mg/kg PIO (Takeda Pharmaceutical Company Limited, Osaka, Japan) or vehicle control (0.5% DMSO) by gavage every day as previously reported [10]. The protocol for the ethics and use of these animals was approved by the Animal Care and Use Committee at Fujita Health University.

At 20 weeks of age, rats were anesthetized with sodium pentobarbital (Schering-Plough Corp., Kenilworth, NJ), and the kidneys were removed rapidly, causing lethal exsanguination. Half of the left kidney was frozen in liquid nitrogen for RNA extraction. Half of the right kidney was immersed in 4% paraformaldehyde, embedded in paraffin, and sectioned for immunohistochemistry.

### 2.2. RNA Extraction

RNA was extracted from kidneys of rats with or without PIO treatment using a monophasic solution of phenol/guanidine isothiocyanate and TRIzol reagent (Invitrogen Co., Carlsbad, CA, USA) in accordance with their manual, and the samples were incubated with RNase-free DNase I (Ambion, TX, USA). The quality and concentration of each sample was confirmed by spectrophotometry (NanoDrop ND-1000; Asahi glass Co. Ltd., Tokyo, Japan). Total RNA obtained from three females was pooled in each PIO-treated or control vehicle-treated (CONT) group in accordance with our previous report [11].

### 2.3. Microarrays

DNA microarray experiments were performed essentially as described previously [11]. Briefly, 500 ng aliquots of total RNA obtained from kidneys of five rats were labeled using a Quick Amp Labeling Kit, one-color (Agilent Technologies, Inc., Santa Clara, CA, USA), according to the manufacturer's instructions. The pooled renal RNA of PIO- or vehicle-treated PCK rats were labeled with the Cy3-fluorescence dye. After determination of labeling efficiency, 1.65 *μ*g aliquots of Cy3-labeled RNA were hybridized using the Gene Expression hybridization kit (Agilent Technologies) onto Rat Oligo Microarrays (Agilent Technologies, product no. G4130A) according to the manufacturer's hybridization protocol. The microarray slides were examined with an Agilent microarray scanner and software. Data analysis was performed with Agilent Feature Extraction software (version A.7.1.1).

Data from microarray experiments of PIO- or vehicle-treated rats were analyzed independently. Primary microarray data are available from the Gene Expression Omnibus (GEO) (accession number GSE00000). Evaluation of signal intensity was divided into three classes, {0}: nondetected, {1}: weakly detected, and {2}: strongly detected transcription product. Gene ontology analysis of biological process (C5BP) and curated gene sets of canonical pathways (C2CP) were analyzed by importing the data into Gene Set Enrichment Analysis (GSEA version 2, the Broad Institute/Massachusetts Institute of technology, USA) [12].

Using the GeneSpring software, the changed probes were listed as “Log 2 ratio was over 1 (over 2-fold) or less than −1 (less than 1/2-fold) between PIO group and CONT group” and “the signal evaluation was {2} (strongly detected) in both groups”. In the changed genes, Kyoto Encyclopedia of Gene and Genomes (KEGG) analysis was used [13].

### 2.4. Real-Time Reverse Transcriptase Polymerase Chain Reaction (RT-PCR)

cDNA was produced from total RNA by reverse transcriptase using random hexamer primers (SuperScript II First Strand Synthesis System; Invitrogen Co., Carlsbad, CA, USA). To compare gene expression patterns of PCK kidneys with PIO or vehicle treatment, we selected a key enzyme in fatty acid metabolism, stearoyl-coenzyme A desaturase 1 (*Scd1*), and uncoupling protein 1 (*Ucp1*). Gene expression was detected by real-time RT-PCR (ABI 7300 real-time PCR system; Applied Biosystems, Foster City, CA, USA) using the TaqMan reagent-based chemistry protocol. Glyceraldehyde-3-phosphate dehydrogenase (*GAPDH*) as a housekeeping gene was used for data normalization. The probes of *Scd1*, *UCP-1, *and *GAPDH* were CCCACATGCTCCAAGAGATCTCCAG, CTCTTCAGGGAGAGAAACGCCTGCC, and AACCCATCACCATCTTCCAGGAGCG, respectively (TaqMan Gene Expression Assays; Applied Biosystems). Relative quantification of gene expression was compared to one in SD control vehicle-treated (CONT) kidneys (set to 1.0).

### 2.5. Immunohistochemistry

Kidney sections were fixed, embedded, and sectioned for immunoreaction as described previously [10, 11]. Sections were incubated with *Scd1* antibody (1 : 250 ab19862 Abcam, Cambridge, UK) in PBS containing 1% BSA plus 0.05% NaN_3_ overnight at 4°C. To test for a specific *Scd1* immunoreaction in the kidney, mouse IgG2b, *κ* isotype control antibody (1 : 200 400323 BioLegend, San Diego, CA), was used. Sections were incubated with secondary antibody Histofine MAX-PO (MULTI: for anti-mouse/rabbit IgG, IgA, and IgM) obtained from Nichirei Biosciences (Tokyo, Japan). Immune reaction products were developed using 3,3′-diaminobenzidine (ENVISION kit HRP Dako Cytomation K3466, Dako Japan Inc., Tokyo, Japan).

### 2.6. Statistical Analysis

Results are expressed as the arithmetic mean ± standard error. Statistical comparisons between groups were performed by Student's *t*-test and two-way analysis of variance, and differences were considered to be significant at *P* < 0.05.

## 3. Results

### 3.1. Identification of Differentially Expressed Genes by Expression Profiling

Previous report indicates that PPAR-*γ* agonistic action decreases expression of endothelin receptor type A (EDNRA) [14], suggesting that *EDNRA* is one of the down-stream target gene of PPAR-*γ* agonists. In our current study, expression of *Ednra* was also downregulated in PIO-treated kidneys (Log2 ratio = −1.30). *EDNRA* expression is increased in human ADPKD, and overexpression of *Ednra* causes cyst formation in transgenic mouse kidneys [15]. Because not only *EDNRA* but also various genes may be influenced by PPAR-*γ* agonistic actions, it became intriguing to determine the expression of other potential gene targets of PIO in PCK rat kidneys. 30,655 of 43,379 probes yielded detectable signals in both PIO- and vehicle-treated kidneys of PCK rats. The 11,809 genes represented by these 30,655 probes were analyzed by GSEA. In gene ontology analysis of biological process (C5BP) gene sets, 334 were formed from these 11,809 genes. 293 of those 334 gene sets were downregulated in PIO-treated kidneys compared with vehicle-treated kidneys, of which 77 were significantly different (*P* < 0.05, Table 1(a)). In the top 20 downregulated C5BP gene sets with the greatest significant differences, 13 were related to cell proliferation, cell cycle, morphogenesis, differentiation, and development, and 4 gene sets were related to cellular defense and inflammation. On the other hand, 41 of the 334 gene sets were upregulated in PIO-treated kidneys compared with vehicle-treated kidneys, of which 6 were significantly different (*P* < 0.05, Table 1(b)). These gene sets were related to catabolic and metabolic processes.

To examine the gene sets with the greatest changes, only 2,611 genes, which changed more than 1.25-fold in PIO-treated kidneys compared to vehicle-treated kidneys, were analyzed. 141 gene sets were formed from these 2,611 genes. 112 of those 141 gene sets were downregulated in PIO-treated kidneys compared with vehicle-treated kidneys of PCK rats. Of these, 6 gene sets were significantly different (*P* < 0.05, Table 2(a)). 4 of these 6 gene sets are related to cell cycle and cell proliferation (Table 2(a)). Common genes in these gene sets include G1/S or G2/M checkpoint related genes, breast cancer 2 (*Brca2*), cyclin-dependent kinase inhibitor 2B (*Cdkn2b*), CHK1 checkpoint homolog (*Chek1*), cell cycle checkpoint protein kinase Bub1 fragment (*BUB1B*), pololike kinase 1 (*PLK1*), and cyclin-dependent kinase inhibitor 1C (*Cdkn1c*) (Table 2(b)). Of the remaining 29 of the 141 gene sets that were upregulated in PIO-treated kidneys compared with vehicle-treated kidneys, only one, related to neurological system processes, was significantly elevated (*P* < 0.05) (Table 2(c)).

In curated gene sets of canonical pathways (C2CP), 257 were formed from the 11,809 genes detected. 201 of these 257 gene sets were downregulated in PIO-treated kidneys compared with vehicle-treated kidneys, of which 33 were significantly lower (*P* < 0.05). From the 20 downregulated C2CP gene sets with the highest significant differences (lowest *P* values), 6 gene sets were related to cell cycle and cell proliferation including c-Jun N-terminal kinase (*JNK*), epidermal growth factor (*EGF*), and platelet-derived growth factor (*PDGF*) pathways, and 3 gene sets were related to inflammatory signals including interleukin-1 receptor (*IL1R*) and interleukin-6 (*IL6*) pathways (Table 3(a)). One gene set, extracellular matrix (ECM) receptor interaction, also was in the top 20 downregulated in C2CP. On the other hand, 56 of 257 gene sets were upregulated in PIO-treated kidneys compared with vehicle-treated kidneys, of which 5 gene sets were significantly higher (*P* < 0.05, Table 3(b)). 3 of these 5 gene sets are related to glutamate, alanine, and aspartate metabolism.

GSEA is a computational method that determines whether an a priori defined set of genes shows statistically significant and concordant differences between two biological states and can detect important biological processes or canonical pathways by using the list rank information without using a threshold [12]. Among the 43,379 probes spotted on the microarray slide, 189 probes were significantly changed. From these 189 probes, 31 genes were identified by KEGG analysis. 23 of those 31 genes were downregulated in PIO-treated compared with vehicle-treated kidneys (Table 4(a)). Two key enzymes in fatty acid metabolism, stearoyl-coenzyme A desaturase 1 (*Scd1*) and uncoupling protein 1 (*Ucp1*), which are involved in PPAR signaling were in the top 15 genes downregulated by PIO treatment. On the other hand, 8 of the 31 genes were upregulated in PIO-treated kidneys compared with vehicle-treated kidneys (Table 4(b)).

### 3.2. Cellular Expression and Distribution of *Scd1* in Rodent Polycystic Kidneys

**Figure 1:**
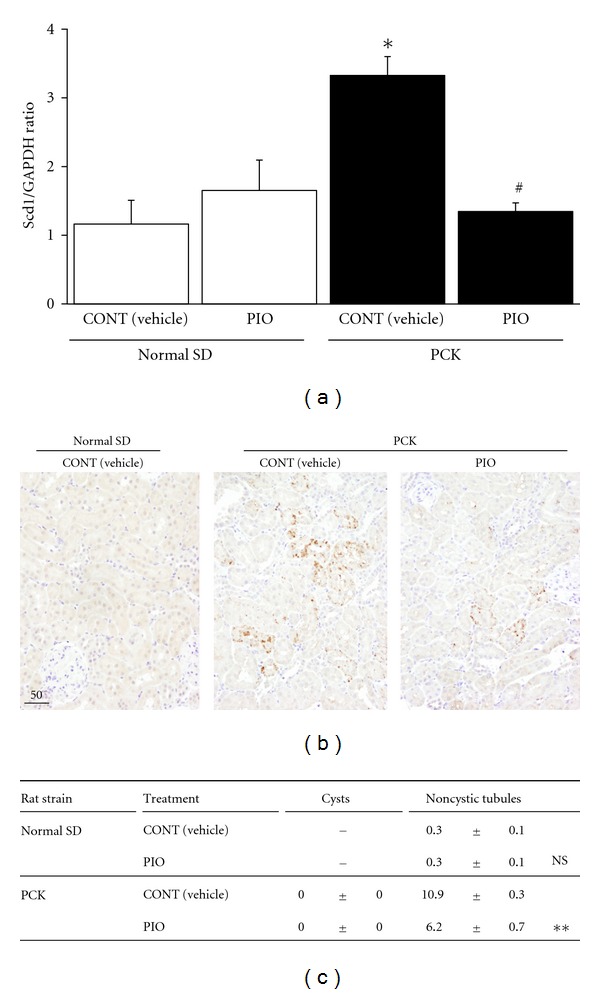
Cellular expression and distribution of *Scd1* in rodent polycystic kidneys. (a) Relative gene expression levels for* Scd1*. mRNA expression levels are shown for vehicle-treted (CONT) or PIO-treated SD and PCK kidneys as compared to vehicle-treated (CONT) SD kidneys (set to 1.0) (*P < 0.05 SD (CONT) versus PCK (CONT), ^#^ *P* < 0.05 PCK (CONT) versus PCK (PIO)). Expression levels were normalized to GAPDH. (b) Renal *Scd1* distribution in vehicle-treated (CONT) or PIO-treated SD and PCK rats. Representative kidney sections from vehicle-treated (CONT) or PIO-treated SD and PCK rats were stained with an antibody to *Scd1*. Mouse IgG2b, *κ* isotype control antibody, did not show any reaction in the kidney. (c) Ratio of *Scd1*-positive cysts or noncystic tubules in kidney sections. Positive-stained cysts or non-cystic tubules were counted in five random fields of kidney sections obtained from five rats in each group by a naive observer using a 20x objective. (**P < 0.01 PCK (CONT) versus PCK (PIO) in noncystic tubules in the kidney section).

For* Scd1* and *Ucp1*, in order to confirm the mRNA expression by DNA microarray screening above, real-time RT-PCR analysis was performed. The mRNA level of *Scd1* in the kidney was increased in PCK rats compared to SD rats and was decreased by PIO treatment in PCK rats (Figure 1(a)). On the other hand, the mRNA level of *Ucp1* was not significantly different between PCK and SD rats (data not shown).

*Scd1* is involved in cell proliferation via growth factors in some type of cancer cells [16–18]. To determine the cellular distribution of *Scd1* in PCK and SD kidneys, immunohistochemistry was used. In normal SD kidneys, *Scd1* was hardly detected. On the other hand, in untreated PCK kidneys, *Scd1* was present in the cytoplasm of normal-shaped tubule epithelia diffusely but not in growing cysts. With PIO treatment, the distribution of *Scd1* decreased in those normal-shaped cells (Figures 1(b) and 1(c)). These findings suggest that *Scd1* may relate to the onset of renal cyst formation originated from normal-shaped tubules.

## 4. Discussion

In our previous report, we demonstrated that PIO treatment in PCK rats inhibited renal Raf/MEK/ERK and AKT/mTOR/S6 activity and reduced proliferation of diseased renal cells [10]. In the current study, we analyzed DNA microarray using GSEA and KEGG pathway analysis in order to detect gene-based effects of PIO treatment [12, 13]. The results of GSEA analysis of C5BP and C2CP are consistent with our previous findings, as a number of gene sets related to cell cycle and cell proliferation are downregulated in kidneys of PIO-treated PCK rats.

Both EGF and PDGF pathways were downregulated by PIO treatment (Table 3(a)). In PKD cystic epithelial cells, growth factors such as EGF and PDGF activate the Raf/MEK/ERK pathway via receptor binding and tyrosine kinase activation [19–21]. Therefore, PIO may ameliorate PKD in PCK rats by inhibiting cell proliferation through suppression of the activity of EGF and PDGF pathways. Further, in PKD patients, several reports show that cystic kidneys have significant levels of apoptosis [22, 23]. The JNK pathway is known to have critical roles in cell apoptosis, and JNK is overexpressed in cystic epithelial cells in Pkd1 conditional knockout mice [23, 24]. In the current study, the JNK MAPK pathway also was downregulated by PIO treatment. Therefore, PIO may have antiapoptotic effects via inactivation of the JNK pathway.

PIO, as well as other PPAR-*γ* agonists rosiglitazone and troglitazone, is known to induce cell cycle arrest and cell apoptosis in human cancer cells [25–27]. Although it has recently been reported that rosiglitazone inhibits cell proliferation by inducing G1 cell cycle arrest in ADPKD cyst-lining epithelial cells [28], the inhibitory mechanism of PIO is under studied in PKD. In the current analysis, *Brca2, BUB1B, Cdkn1c, Cdkn2b, Chek1, *and* PLK1* were downregulated. These genes are involved in cell cycle regulation, G0/G1, G1/S and/or G2/M checkpoints [29–35], suggesting that the antiproliferative effect of PIO may be related to cell cycle arrest.

After searching each gene expression with significant change by PIO treatment, we then focused on *Scd1* because it is known to stimulate cell proliferation in cancer cells through phosphorylation of AKT [16–18], one of the responsible kinases in cystic cell proliferation in PKD [10, 36]. Immunohistochemical analysis demonstrated that *Scd1* expression was increased in noncystic tubules in PCK kidneys, and PIOtreatment reduced its overexpression, suggesting that *Scd1* may relate to the onset of cell proliferation in initial cyst formation through phosphorylation of AKT. In addition, activation of the cell cycle increases syntheses of phospholipids and cholesterol [37–39], and *Scd1* controls the balance of saturated and monounsaturated fatty acids, regulating the composition of cholesterol esters and phospholipids in cell membrane structure [16]. Therefore, PIO may reduce cell proliferation by the downregulation of *Scd1* gene expression not only through reducing AKT signaling activity but also through altering fatty acid synthesis. In abnormal cell proliferation in cancer, *Scd1* expression is increased, and the cell proliferation is suppressed by treatment with PPAR-*γ* agonists, although the changes in *Scd1* expression are not always consistent [16, 40, 41]. On the other hand, in diabetes mellitus with insulin resistance, adipose tissue or skeletal muscle *Scd1* expression is decreased and increased by PPAR-*γ* agonists [42–44]. Therefore, the expression level of *Scd1* and the effect of PPAR-*γ* agonists may depend on the disease and/or the state of cell proliferation.

Clinically, increased body weight, oedema, and urinary bladder tumors are concerned as possible side effects of PPAR-*γ* agonists. Although those phenomena were not observed in both genders of PCK rats in the current PIO treatment, the effect of longer term treatment with different doses will need to be studied carefully. Since ameliorative effects are reported in several animal models of PKD [8–10, 45], PPAR-*γ* agonists are thought to be a potential candidate for therapeutic interventions in both ARPKD and ADPKD patients.

## 5. Conclusions

In the current study, PIO reduced PKD progression and altered the expression of renal genes involved in cell proliferation, cell cycle progression, and fatty acid metabolism in an orthologous rat model of human ARPKD. In addition to the previously demonstrated inhibition of Raf/MEK/ERK and AKT/mTOR/S6 signaling pathways by treatment of PCK rats with 10 mg/kg PIO for 16 weeks [10], suppression of cell proliferation may also be related to reductions in EGF, PDGF, and JNK pathways, cell cycle arrest related to *Brca2, BUB1B*, *Cdkn1c*, *Cdkn2b*, *Chek1,* and *PLK1* genes, and alteration of fatty acid metabolism related to *Scd1*.

## Figures and Tables

**Table 1a:** (a)

Name of biological process gene sets	Number of genes in the gene set	Nominal *P* value
Defense response	98	0.000
Regulation of cell proliferation	136	0.000
Cell cycle phase	53	0.000
Positive regulation of cell proliferation	64	0.000
Cell cycle process	61	0.000
Positive regulation of cellular process	258	0.000
Cellular morphogenesis during differentiation	22	0
Positive regulation of developmental process	91	0.001
Immune system process	128	0.001
Cellular defense response	19	0.003
Neuron differentiation	35	0.004
Negative regulation of cell proliferation	73	0.004
Neurite development	27	0.005
G Protein signaling coupled to ip3 second messenger phospholipase C activating	22	0.005
Inflammatory response	56	0.005
Regulation of response to stimulus	15	0.006
Neuron development	30	0.007
M phase	27	0.007
Interphase	29	0.008
Axonogenesis	21	0.009

**Table 1b:** (b)

Name of biological process gene sets	Number of genes in the gene set	Nominal *P* value
Nitrogen compound catabolic process	17	0.000
Amine catabolic process	15	0.000
Amino acid metabolic process	46	0.000
Amino acid and derivative metabolic process	58	0.000
Organic acid metabolic process	106	0.000
Carboxylic acid metabolic process	104	0.022

**Table 2a:** (a)

Name of biological process gene sets	Number of genes in the gene set	Nominal *P* value
Carbohydrate METABOLIC PROCESS	16	0.019
Cell proliferation GO 0008283	70	0.024
Organelle organization and biogenesis	34	0.025
Cell cycle GO 0007049	44	0.027
Negative regulation of cell proliferation	28	0.032
Cell cycle process	31	0.037

**Table 2b:** (b)

Gene symbol	Description	Name of biological process gene sets			
		Cell cycle GO 0007049	Negative regulation of cell proliferation	Cell cycle process	Cell proliferation GO 0008283
*Brca2*	Breast cancer 2	*✓*	*✓*	*✓*	*✓*
*Cdkn2b*	Cyclin-dependent kinase inhibitor 2B (p15, inhibits CDK4)	*✓*	*✓*	*✓*	*✓*
*Chek1*	CHK1 checkpoint homolog	*✓*	*✓*	*✓*	—
*BUB1B*	Cell cycle checkpoint protein kinase Bub1 Fragment	*✓*	*✓*	—	*✓*
*PLK1*	Pololike kinase 1	*✓*	*✓*	—	*✓*
*Cdkn1c*	Cyclin-dependent kinase inhibitor 1C	*✓*	*✓*	*✓*	—
*Cul5*	Cullin 5	*✓*	*✓*	*✓*	*✓*
*Tgfb2*	Transforming growth factor, beta 2	*✓*	—	*✓*	*✓*
*Bcat1*	Branched chain aminotransferase 1	*✓*	*✓*	—	*✓*
*PTPRC*	Protein tyrosine phosphatase, receptor type, C	*✓*	*✓*	—	*✓*
*POLA1*	Polymerase (DNA directed), alpha 1	*✓*	*✓*	—	*✓*

**Table 2c:** (c)

Name of biological process gene sets	Number of genes in the gene set	Nominal *P* value
Neurological System Process	40	0.032

**Table 3a:** (a)

Name of biological process gene sets	Number of genes in the gene set	Nominal *P* value
HSA04640 hematopoietic cell lineage	32	0.000
HSA04610 complement and coagulation cascades	37	0.000
HSA04510 focal adhesion	110	0.001
Breast cancer estrogen signaling	60	0.001
HSA04060 cytokine cytokine receptor interaction	99	0.002
HSA04912 GNRH Signaling Pathway	64	0.002
HSA04110 cell cycle	44	0.003
HSA01430 cell communication	39	0.004
IL1R pathway	15	0.007
Eicosanoid synthesis	15	0.009
HSA04512 ECM receptor interaction	41	0.009
Cell cycle KEGG	34	0.012
ST JNK MAPK pathway	17	0.017
EGF pathway	23	0.023
PDGF pathway	23	0.028
FCER1 pathway	26	0.029
GSK3 pathway	18	0.029
Prostaglandin and leukotriene metabolism	19	0.032
IL6 pathway	17	0.032
HSA02010 ABC transporters general	21	0.033

**Table 3b:** (b)

Name of biological process gene sets	Number of genes in the gene set	Nominal *P* value
HSA00190 oxidative phosphorylation	37	0.000
Glutamate metabolism	15	0.000
HSA00252 alanine and aspartate metabolism	17	0.010
HSA00710 carbon fixation	15	0.019
HSA00251 glutamate metabolism	17	0.019

**Table 4a:** (a)

Gene symbol	Description	KEGG pathway	PIO/CONT Log 2 ratio
*Olr1436*	Olfactory receptor 1436	Olfactory transduction	−3.27
*Xylt1*	Xylosyltransferase 1	Glycosaminoglycan biosynthesis-chondroitin sulfate/glycosaminoglycan biosynthesis-heparan sulfate/metabolic pathways	−3.03
*Map3k10*	Mixed-lineage kinase 2	MAPK signaling pathway	−2.24
*Icoslg*	—	Cell adhesion molecules (CAMs)/intestinal immune network for IgA production	−2.15
*Scd1*	Stearoyl-coenzyme A desaturase 1	Biosynthesis of unsaturated fatty acids/PPAR signaling pathway	−2.01
*Ucp1*	Uncoupling protein 1	PPAR signaling pathway/Huntington's disease	−1.91
*Oxt*	Oxytocin, prepropeptide	Neuroactive ligand-receptor interaction	−1.81
*Chrm1*	Cholinergic receptor, muscarinic 1	Calcium signaling pathway/Neuroactive ligand-receptor interaction/regulation of actin cytoskeleton	−1.75
*Avp*	Arginine vasopressin	Neuroactive ligand-receptor interaction/vascular smooth muscle contraction/vasopressin-regulated water reabsorption	−1.58
*Lpcat2*	Lysophosphatidylcholine acyltransferase 2	Glycerophospholipid metabolism/ether lipid metabolism/metabolic pathways	−1.37
*Il12rb1*	Interleukin 12 receptor, beta 1	Cytokine-cytokine receptor interaction/jak-STAT signaling pathway	−1.34
*EDNRA*	Endothelin receptor type A	Calcium signaling pathway/neuroactive ligand-receptor interaction/vascular smooth muscle contraction	−1.30
*Cfd*	Complement factor D (adipsin)	Complement and coagulation cascades	−1.20
*Serpinb5*	Serine (or cysteine) peptidase inhibitor, clade B, member 5	p53 signaling pathway	−1.19
*Htr2b*	5-Hydroxytryptamine (serotonin) receptor 2B	Calcium signaling pathway/neuroactive ligand-receptor interaction/gap junction	−1.19
*Cox8b*	Cytochrome c oxidase, subunit VIIIb	Oxidative phosphorylation/metabolic pathways/cardiac muscle contraction/Alzheimer's disease/Parkinson's disease/Huntington's disease	−1.17
*Peg12*	Paternally expressed 12	Wnt signaling pathway	−1.11
*Sema3d*	Sema domain, immunoglobulin domain (Ig), short basic domain, secreted, (semaphorin) 3D	Axon guidance	−1.07
*Atp1a2*	ATPase, Na+/K+ transporting, alpha 2 polypeptide	Cardiac muscle contraction/aldosterone-regulated sodium reabsorption/proximal tubule bicarbonate reclamation/salivary secretion/gastric acid secretion	−1.05
*Dll3*	Delta-like 3	Notch signaling pathway	−1.05
*Brca2*	Breast cancer 2	Homologous recombination/pathways in cancer/pancreatic cancer	−1.04
*Aqp4*	Aquaporin 4 (Aqp4), transcript variant 2	Vasopressin-regulated water reabsorption	−1.02
*Gys2*	Glycogen synthase 2	Starch and sucrose metabolism/insulin signaling pathway	−1.01

KEGG pathway: Koto Encyclopedia of Gene and Genomes pathway.

**Table 4b:** (b)

Gene symbol	Description	KEGG pathway	PIO/CONT Log 2 ratio
*Gucy2d*	Guanylate cyclase 2d (Gucy2d)	Purine metabolism/olfactory transduction/phototransduction	1.59
*Cyp2b1*	Cytochrome P450, family 2, subfamily b, polypeptide 1 (Cyp2b1), mRNA	Arachidonic acid metabolism/retinol metabolism/metabolism of xenobiotics by cytochrome P450/drug metabolism-cytochrome P450/metabolic pathways	1.45
*Cyp2d3*	Cytochrome P450, family 2, subfamily d, polypeptide 3 (Cyp2d3)	Drug metabolism-cytochrome P450	1.20
*Tarsl2*	Threonyl-tRNA synthetase-like 2 (Tarsl2), mRNA	Aminoacyl-tRNA biosynthesis	1.17
*Prl*	Prolactin (Prl), mRNA	Cytokine-cytokine receptor interaction/neuroactive ligand-receptor interaction/jak-STAT signaling pathway	1.17
*Olr1331*	Olfactory receptor 1331 (Olr1331), mRNA	Olfactory transduction	1.17
*Dync1h1*	Dynein cytoplasmic 1 heavy chain 1 (Dync1h1), mRNA	Phagosome/vasopressin-regulated water reabsorption	1.11
*Olr297*	Olfactory receptor 297 (Olr297)	Olfactory transduction	1.06

KEGG pathway: Koto Encyclopedia of Gene and Genomes pathway.
